# Therapeutic implications of selecting the SCORE (European) versus the D'AGOSTINO (American) risk charts for cardiovascular risk assessment in hypertensive patients

**DOI:** 10.1186/1471-2261-9-17

**Published:** 2009-05-11

**Authors:** Manuel A Gómez-Marcos, Carlos Martínez-Salgado, Carlos Martin-Cantera, José I Recio-Rodríguez, Yolanda Castaño-Sánchez, Maria Giné-Garriga, Emiliano Rodriguez-Sanchez, Luis García-Ortiz

**Affiliations:** 1 Primary care Research Unit, La Alamedilla Health Centre, REDIAPP, Salamanca, Spain; 2 Research Unit, Salamanca University Hospital, Unit of Renal and Cardiovascular Pathophysiology, Reina Sofia Institute of Nephrological Research, REDNIREN, Salamanca, Spain; 3 Research Support Unit, IDIAP Jordi Gol, REDIAPP, Passeig de Sant Joan Primary Care Centre, Barcelona, Spain; 4 Physical Activity and Health area, Catalan Institute of Health, FPCEE Blanquerna, Ramon Llull University, Barcelona, Spain

## Abstract

**Background:**

No comparisons have been made of scales estimating cardiovascular mortality and overall cardiovascular morbidity and mortality. The study objectives were to assess the agreement between the Framingham-D'Agostino cardiovascular risk (CVR) scale and the chart currently recommended in Europe (SCORE) with regard to identification of patients with high CVR, and to describe the discrepancies between them and the attendant implications for the treatment of hypertension and hyperlipidaemia.

**Methods:**

A total of 474 hypertensive patients aged 40–65 years monitored in primary care were enrolled into the study. CVR was assessed using the Framingham-D'Agostino scale, which estimates the overall cardiovascular morbidity and mortality risk, and the SCORE chart, which estimates the cardiovascular mortality risk. Cardiovascular risk was considered to be high for values ≥ 20% and ≥ 5% according to the Framingham-D'Agostino and SCORE charts respectively. Kappa statistics was estimated for agreement in classification of patients with high CVR. The therapeutic recommendations in the 2007 European Guidelines on Cardiovascular Disease Prevention were followed.

**Results:**

Mean patient age was 54.1 (SD 7.3), and 58.4% were males. A high CVR was found in 17.5% using the SCORE chart (25.3% males, 6.6% females) and in 32.7% using the D'Agostino method (56.9% males, 12,7% females). Kappa coefficient was 0.52, and increased to 0.68 when the high CVR threshold was established at 29% according to D'Agostino. Hypertensive patients with high SCORE and non-high D'Agostino (1.7%) were characterized by an older age, diabetes, and a lower atherogenic index, while the opposite situation (16.9%) was associated to males, hyperlipidaemia, and a higher atherogenic index. Variables with a greater weight in discrepancies were sex and smoking. A 32.0% according to SCORE and 33.5% according to D'Agostino would be candidates to receive antihypertensive treatment, and 15.8% and 27.3% respectively to receive lipid-lowering treatment.

**Conclusion:**

A moderate to high agreement was found. SCORE may underestimate risk in males with an unfavourable lipid profile, and D'Agostino in diabetics with a lower atherogenic risk. Use of the D'Agostino scale implies treating more patients with lipid-lowering and antihypertensive drugs as compared to SCORE.

## Background

Arterial hypertension (AH) is the most prevalent cardiovascular risk (CVR) factor and the leading cause of morbidity and mortality from cardiovascular disease in Spain. AH is related to 46.4% of deaths from cerebrovascular disease and to 42% of deaths from heart disease[1,2].

Current clinical guidelines recommend estimation of cardiovascular risk in patients with hypertension and dyslipidaemia to graduate treatment intensity and thus optimise cost-effectiveness [3-5].

Both the European Guidelines on Cardiovascular Disease Prevention and the Interdisciplinary Spanish Committee for Cardiovascular Prevention (CEIPC)[6] recommend use of the SCORE (Systematic Coronary Risk Evaluation) charts[7], which estimate the risk of death from cardiovascular disease, although their calibration in Spain has shown that they underestimate the risk of cardiovascular morbidity and mortality[8]. A new scale based on the Framingham Heart Study (D'Agostino)[9] intended to estimate the risk of morbidity and mortality from cardiovascular disease has recently been published.

A review of the multiple studies published in recent years reveals that there is no ideal scale for assessing CVR in Mediterranean populations. The original Framingham function[10,11] in its different versions, identifies more patients with a high CVR than the SCORE chart[12,13]. However, its calibration for the Spanish population (REGICOR)[14,15] identifies less patients with a high CVR than the original scale[16,17], and agreement in identification of patients with high CVR is low [18-21]. It should be kept in mind that scales derived from the Framingham function estimate the incidence of coronary artery disease, except for the D'Agostino scale, which estimates overall cardiovascular morbidity and mortality, whereas SCORE estimates cardiovascular mortality. The Framingham scales are commonly used as a standard to assess other methods, such as the presence of calcium in the coronary artery[22], and also for assessing the effectiveness of certain interventions[23]. However, no analysis has been made of agreement between the SCORE and D'Agostino scales and of the patient subgroups in which they disagree. The attendant therapeutic implications in patients with hypertension and hypercholesterolaemia have not been studied either.

Therefore, the aims of this study were to assess the agreement between these scales in hypertensive patients aged 40–65 years, to describe patient characteristics that are associated to disagreement in identification of patients with a high CVR, and to assess the impact of both functions on therapeutic indications for blood pressure and hypercholesterolaemia.

## Methods

### Study population

Patients with a clinical diagnosis of arterial hypertension from to urban health care centres covering a population of 46.000 inhabitants. For this analysis, 474 patients with clinical hypertension referred to the research unit for confirmation of diagnosis by ambulatory blood pressure monitoring (ABPM) or evaluation of target organ lesions in cases with documented hypertension were consecutively enrolled from December 2005 to June 2008. Patients had to meet the following inclusion criteria: (a) age ranging from 40 and 65 years, and (b) no history of established cardiovascular disease. Variables analysed included all those required to estimate CVR using both scales.

### Measurements of risk factors and cardiovascular risk

Risk factors for morbidity and mortality used by the D'Agostino scale [9] include age, sex, total cholesterol, high-density lipoprotein cholesterol (HDL-C), and systolic blood pressure (SBP) as quantitative variables, and drug treatment for AH, smoking, and history of diabetes mellitus as dichotomous variables. Risk of cardiovascular morbidity and mortality was estimated using the published risk equation based on the Framingham study[9].

Cardiovascular death risk variables used by SCORE include age, sex, total cholesterol, SBP, and smoking as dichotomous variable. These tables do not include diabetes as a CVR factor, but authors recommend multiplication by 4 in females and by 2 in males if diabetes is found, and this was the option adopted in our study. To estimate cardiovascular risk with SCORE, the version for low risk countries, using total cholesterol from the risk equation based on European cohorts, has been used[7]. The following variables were also collected: blood glucose, full lipid profile, body mass index (BMI), and use of lipid lowering drugs.

Measurements were performed by nursing staff at the research unit. Blood pressure was measured using OMROM M7^® ^sphygmomanometers in accordance with the recommendations by the European Society of Hypertension[24]. Lipid and blood glucose levels were measured blindly at the reference laboratory after fasting for at least 8 hours. Data on CVR factors were taken from the clinical history of each patient. The study was approved by independent ethics committee from University Hospital of Salamanca (Spain), and all participants signed an informed consent.

### Patient classification according to risk

Patients with a risk of cardiovascular morbidity and mortality ≥ 20% in the Framingham-D'Agostino scales and a risk of cardiovascular death ≥ 5% in the SCORE charts were considered to have a high CVR. A CVR ranging from 6%–20% according to D'Agostino and from 3%–4.9% according to SCORE was considered as moderate, and a risk < 6% in the D'Agostino scale and < 3% in the SCORE scale was considered as low, in agreement to recommendations published by the authors[7,9].

The recommendations in the 2007 European Guidelines on Cardiovascular Disease Prevention were applied to estimate the proportion of patients amenable to drug treatment (antihypertensive or lipid lowering). Candidates to receive antihypertensive drug treatment include patients with SBP ≥ 160 mmHg or DBP ≥ 100 mmHg, irrespective of their CVR, patients with SBP ≥ 140 mmHg or DBP ≥ 90 mmHg and a CVR ≥ 5% according to SCORE or ≥ 20% according to D'Agostino or diabetics, and patients with SBP ranging from 140–159 mmHg or DBP ranging from 90–99 mmHg and a moderate CVR according to SCORE or D'Agostino. With regard to plasma lipid control, patients with total cholesterol > 320 mg/dL and LDL-C > 240 mg/dL, diabetes or a CVR ≥ 5% according to SCORE or ≥ 20% according to D'Agostino, and total cholesterol ≥ 190 mg/dL and/or LDL-C ≥ 115 mg/dL are considered to be candidates to drug treatment. To perform calculations, antihypertensive or lipid lowering treatments were considered to be adequately indicated in subjects who were already receiving them[12,13,25].

### Statistical analysis

Characteristics of patients studied and the CVR levels were described using measures of central tendency and dispersion for quantitative variables and percentages for categorical variables. These measures were compared between different subgroups using a Student's t test for quantitative variables and qualitative variables of two categories, and a Chi-square test for qualitative variables. kappa coefficient was used to assess agreement of both scales in study subjects. Agreement was considered "excellent" when values ranging from 0.81–1 were obtained, "good" when values ranged from 0.61–0.80, and "moderate" when values of 0.41–0.60 were found[26], and a Pearson's correlation was applied for quantitative measurement. In order to assess the association between patient characteristics and disagreement in categorisation as high risk for both scales, a multivariate analysis was performed using the forward stepwise logistic regression method, taking as dependent variable the discrepancies between the two equations, and including age, sex, smoking, present or absent diabetes, basal blood glucose, total cholesterol, HDL-C, atherogenic index, SBP, and DBP as independent variables. To assess the diagnostic accuracy of SCORE as compared to the different cut-off points of high CVR in the D'Agostino scale, the area under the ROC curve was used. A value of p ≤ 0.05 was considered statistically significant. All analyses were performed using SPSS/PC+ version 15.0 software.

## Results

### Study population

Among the total 914 hypertensive patients seen at the research unit during the study period, 474 (52%) were within the recommended age range for use of scales and had no history of cardiovascular disease. Table 1 shows blood pressure values and levels of different biochemical markers, as well as sex differences in patients participating in the study.

**Table 1:** Characteristics of study patients, overall and by sex.

	Overall (n=74)	Males **(**n = 277**)**	Females **(**n = 197**)**	p
Age (years)	54.10 ± 7.27	54.09 ± 7.49	54.12 ± 6.97	0.962
Obese (BMI > 30)	153 (32.3%)	92 (33.2%)	61 (31.0%)	0.606
Smokers	111 (23.4%)	69 (24.9%)	42 (21.3%)	0.363
Diabetics	73 (15.4%)	52 (18.8%)	21 (10.7%)	0.016
Hyperlipidaemic	314 (66.2%)	174 (62.8%)	140 (71.1%)	0.038
BMI	28.72 ± 4.71	28.91 ± 4.14	28.46 ± 5.41	0.310
Waist circumference	96.04 ± 12.38	100.10 ± 9.99	90.28 ± 13.15	0.000
Glucose (mg/dL)	100.28 ± 28.04	103.32 ± 30.76	95.94 ± 23.00	0.006
Cholesterol/HDL ratio	4.12 ± 1.09	4.38 ± 1.12	3.76 ± 0.94	0.000
Total cholesterol (mg/dL)	214.70 ± 35.92	211.70 ± 36.17	218.92 ± 35.22	0.031
HDL-C (mg/dL)	54.85 ± 14.44	50.54 ± 12.34	60.92 ± 15.01	0.000
LDL-C (mg/dL)	134.18 ± 32.99	134.37 ± 32.46	133.92 ± 33.83	0.888
Triglycerides (mg/dL)	115.26 ± 73.81	137.79 ± 80.32	128.38 ± 61.47	0.001
Systolic blood pressure (mmHg)	140.99 ± 18.92	142.86 ± 16.49	138.37 ± 21.65	0.011
Diastolic blood pressure (mmHg)	89.09 ± 10.40	89.70 ± 9.67	88.24 ± 11.32	0.132
Antihypertensive drugs	271 (57.2%)	159 (57.4%)	112 (56.9%)	0.905
Lipid lowering drugs	75 (15.8%)	52 (18.8%)	23 (11.7%)	0.037
CVR by SCORE*	2.97 ± 4	3.94 ± 4.66	1.61 ± 2.18	0.000
CVR by D'Agostino**	18.00 ± 13.45	22.88 ± 14.66	11.12 ± 7.22	0.000

Hyperlipidaemic: cholesterol > 200 mg/dl. BMI: body mass index; HDL: high-density lipoprotein; HDL-C: high density lipoprotein cholesterol; LDL-C: low density lipoprotein cholesterol. CVR: cardiovascular risk. Significant differences between males and females. P value: Differences between males and females. Data are shown as mean ± standard deviation or as number and percentages. *Cardiovascular mortality risk as percent risk and estimated at 10 years. **Cardiovascular morbidity and mortality risk as percent risk and estimated at 10 years.

### Estimated cardiovascular risk

Mean CVR was 18% (95% CI 16.78–19.21) according to D'Agostino and 2.97% (95% CI 2.61–3.33) according to SCORE, with a Pearson's correlation coefficient of 0.836 (p = 0.000) between both scales.

**Figure 1:**
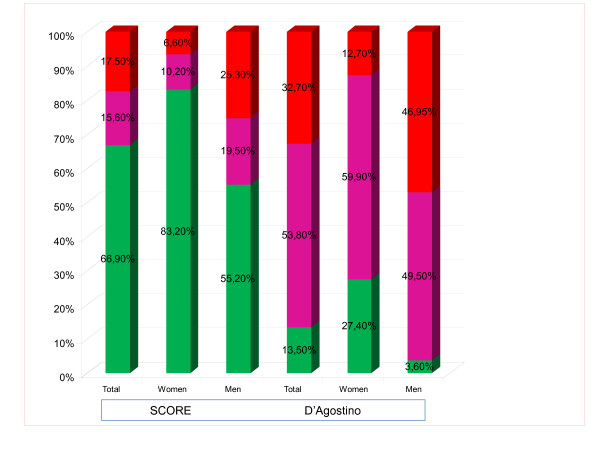
**Distribution (in percentages) of the population in risk categories, overall and by sex, in the D'AGOSTINO and SCORE functions**.

The proportions of patients considered to have a high CVR were 17.5% with SCORE (25.3% in males and 6.6% in females) and 32.7% with D'Agostino (46.9% in males and 12.7% in females) (p < 0.05). Figure 1 shows the proportions of patients included in the low, moderate, and high CVR categories, both overall and by sex.

Table 2 shows distribution of the population based on the presence of a high or non-high CVR, using high risk cut-off points of 20% for the D'Agostino scale and 5% for SCORE. Disagreement was found in 18.57% of patients (high CVR in one chart and non-high CVR in the other chart). Of these, 16.88% had a high CVR according to D'Agostino and a non-high CVR with SCORE, while the opposite occurred in the remaining 1.69%. However, when the 163 patients rated as having a high CVR by any scale were only analysed, the disagreement rate increased to 54% of cases.

**Table 2:** Classification of cardiovascular risk in the high and non-high categories. Agreement in the high and low risk categories.

**Assessment by SCORE**	**Assessment by D'Agostino**	
	Non-high CVR	High CVR
Non-high CVR	311 (65.60%)	80 (16.88%)
High CVR	8 (1.69%)	75 (15.82%)

CVR: cardiovascular risk. Data are shown as n (%) of the total population analysed. Kappa: 0.52 (95% CI, 0.48–0.56); McNemar's test χ^2 ^= 152.01. p < 0.001; discrepancies 18.57%.

**Figure 2:**
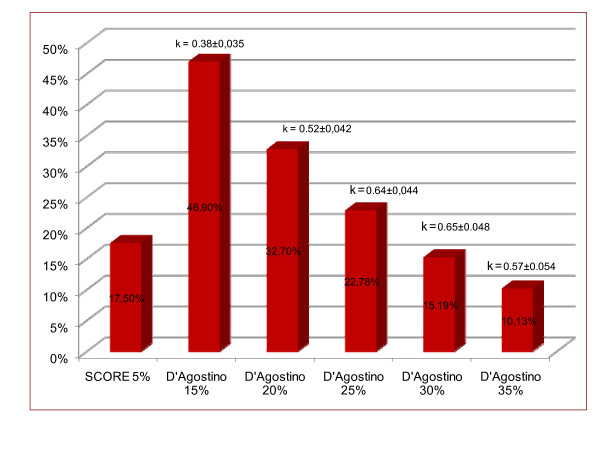
**Proportions of subjects classified as high risk with SCORE (5%) and D'AGOSTINO at different cut-off points and agreement with SCORE**. κ: Kappa index.

Figure 2 shows the percentages of subjects with high CVR according to D'Agostino for different cut-off points, with their kappa indexes as compared to SCORE. The kappa index was higher as the high CVR cut-off point in the D'Agostino scale increased, with the greatest agreement being found in the cut-off point of 29% (kappa 0.678 [95% CI, 0.632–0.724]), in which 90.93% of patients were classified in the same way using both scales.

**Figure 3:**
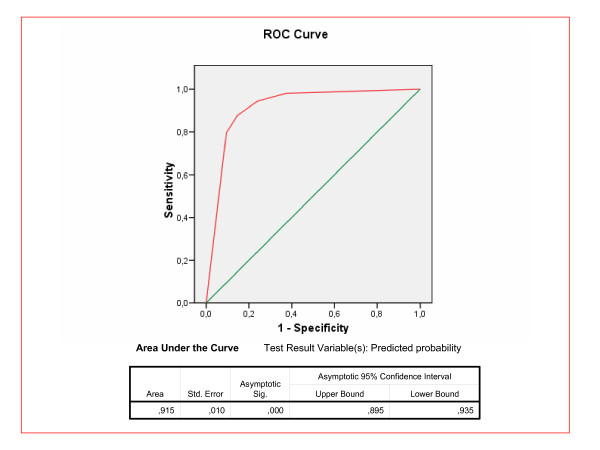
**ROC curve for the D'Agostino scale versus SCORE 5%**. The test result variable(s): Predicted probability has at least one tie between the positive actual state group and the negative actual state group.

Figure 3 shows the discriminating power, calculated using the ROC curve between 5% in SCORE and different cut-off points for high CVR according to D'Agostino. The area under the curve was 0.915. The optimum cut-off point in the D'Agostino scale was 25% (sensitivity 88% and specificity 86%). At this threshold, disagreement in classification of patients with high CVR with the D'Agostino scale as compared to SCORE was only 11.6%.

### Analysis of discordant patients

Most disagreeing patients, i.e., hypertensive patients rated as high CVR in a scale and not high CRV in the other, correspond to the group with a high CVR in the D'Agostino scale and not high in the SCORE scale. This group show a greater proportion of males (80% versus 50%), higher mean LDL-C levels (141 versus 107 mg/dL), and a higher proportion of patients treated with antihypertensive drugs (77% versus 37%). Only eight hypertensive patients were rated as high CVR with the SCORE scale and not high with the D'Agostino scale (Table 3).

**Table 3:** Characteristics of patients with discordant results using the SCORE and D'Agostino equations.

	SCORE high-D'AGOSTINO non-high (n = 8)	SCORE non-high-D'AGOSTINO high (n = 80)	p
Males	4 (50%)	64 (80%)	0.054
Age (years)	62.69 ± 8.83	56.41 ± 5.6	0.003
Obese (BMI > 30)	3 (37.5%)	27 (33.8%)	0.831
Smokers	1 (12.5%)	29 (36.3%)	0.177
Diabetics	5 (62.5%)	17 (21.3%)	0.010
Hyperlipidaemic	2 (25%)	55 (68.8%)	0.014
BMI	31.6 ± 7.53	28.70 ± 4.40	0.102
Waist circumference	97.5 ± 9.77	99 ± 10.39	0.697
Glucose (mg/dL)	110.50 ± 40.59	105.82 ± 31.09	0.694
Cholesterol/HDL ratio	3.06 ± 0.81	4.76 ± 1.08	0.000
Total cholesterol (mg/dL)	196.50 ± 43.39	219.79 ± 36.39	0.093
HDL-C (mg/dL)	68.25 ± 25.10	47.64 ± 10.36	0.000
LDL-C (mg/dL)	107.13 ± 27.46	140.78 ± 34.01	0.008
Triglycerides (mg/dL)	105 ± 55.10	155.55 ± 96.74	0.151
Systolic blood pressure (mmHg)	137.75 ± 18.27	146.43 ± 17.87	0.195
Diastolic blood pressure (mmHg)	83.63 ± 9.33	91.11 ± 10.97	0.066
Antihypertensive drugs	3 (37.5%)	62 (77.5%)	0.014
Lipid lowering drugs	2 (25%)	20 (25%)	1

BMI: body mass index; Hyperlipidaemic: cholesterol > 200 mg/dl, HDL: high-density lipoprotein; HDL-C: high density lipoprotein cholesterol; LDL-C: low density lipoprotein cholesterol. P value of the association of the typical differences and the probability of being a discordant patient (SCORE non-high-D'AGOSTINO high; SCORE high-D'AGOSTINO non-high). Data are shown as mean ± standard deviation or as number and percentages.

Table 4 shows the variables independently associated to discrepancies. Variables with greater odds ratios included sex (being male) (2.45) and smoking (1,86).

**Table 4:** Variables associated to discrepancies in classification as high CVR

	Odds Ratio (95% CI)	p
Age	1.089 (1.048–1.132)	0.000

Sex (1 = Male/0 = Female)	2.465 (0.384–4.390)	0.002

Smoker (1 = yes/0 = no)	1.858 (1.052–3.282)	0.033

Atherogenic index	1.475 (1.174–1.853)	0.001

M: Male; F: Female.

Dependent variable: 1 = (Discrepancy in classification between both scales); 0 = (Agreement in classification between both scales). Multivariate analysis (logistic regression). Atherogenic index = Total Cholesterol/HDL-Cholesterol. Significant model (χ^2 ^= 54.285; p = 0.000).

### Therapeutic implications

Compliance with the recommendations in the European Guidelines on Cardiovascular Disease Prevention would imply that 32% of patients without treatment, according to the SCORE function, and 33.5% of patients according to the D'Agostino chart would be candidates to receive antihypertensive drugs, and 15.8% and 27.3% of patients without treatment respectively would be candidates to receive lipid lowering drugs. Lipid lowering and antihypertensive drugs would be indicated in a significantly higher percentage of males as compared to females (Table 5).

**Table 5:** Patients in the total population candidates to drug treatment (lipid lowering or antihypertensive) according to recommendations in the European Guidelines on Cardiovascular Disease Prevention using the SCORE and D'Agostino equations.

	With SCORE	With D'Agostino	p
Antihypertensive treatment			
Overall	65 (32%)	68 (33.5%)	0.453
Males	48 (40.7%)	51 (43.2%)	0.453
Females	17 (20%)	17 (20%)	1
			
Lipid lowering treatment			
Overall	63 (15.8%)	109 (27.3%)	0.000
Males	51 (22.7%)	89 (39.6%)	0.000
Females	12 (6.9%)	20 (11.5%)	0.008

P value of differences in treatment based on European Guidelines on Cardiovascular Disease Prevention between both scales.

## Discussion

In this study on hypertensive patients aged 40–65 years with no history of cardiovascular disease, agreement in identification of patients with high CVR between the SCORE equation[7] for low-risk countries using a cut-off point of 5% and the D'Agostino equation[9] using a cut-off point of 20% was moderate (kappa = 0.52), and increased when the cut-off point in this second scale was increased to 29% (kappa = 0.68).

The population with high CVR according to SCORE and a non-high CVR according to D'Agostino consisted of older patients with diabetes and a lower atherogenic index, while a greater proportion of males with a higher atherogenic index and on antihypertensive treatment were found in the group with high CVR using the SCORE equation and non-high CVR using the D'Agostino equation. SCORE may underestimate CVR in males with an unfavourable lipid profile and treated with drugs for AH as compared to the D'Agostino method. By contrast, the D'Agostino equation may underestimate CVR in elderly patients with diabetes and higher HDL-C levels as compared to the SCORE chart.

As regards therapeutic decisions, use of the D'Agostino classification of CVR implies treating 3.5% more males with antihypertensive drugs and 11.6% more patients with lipid lowering drugs (mainly at the expense of males) as compared to when the SCORE equation is used. Thus, if SCORE is used, we will leave untreated a significant number of males with high CVR according to D'Agostino, a group in which clinical trials have shown a greater efficacy of lipid lowering treatment for prevention of ischaemic heart disease.

The proportion of patients rated as having a high CVR using the SCORE equation was higher than reported in other studies seen in primary care [18,20] and in the general population[12,21], and similar to the proportions reported by studies on patients with hypertension or hyperlipidaemia[19,27]. In this study, as in other studies in similar populations[19,21,27], most female patients were considered to have a low CVR using the SCORE equation. By contrast, most patients analysed, both males and females, were considered to have a moderate CVR risk when the D'Agostino scale was used. Almost twice as much patients are classified as having a high CVR with the D'Agostino as compared to the SCORE equation, and both scales assign a high CVR to a much greater proportion of males, 4 males per each female.

These data suggest that the scale derived from the D'Agostino equation overestimates CVR as compared to SCORE, despite the fact that it calculates CVR instead of only coronary risk, as occurred to date with the different versions derived from the Framingham equation. This scale would therefore have to be calibrated in our population, or a higher cut-off point would have to be established to classify the same percentage of patients as having a high CVR. Such cut-off point would be from 25% to 30%, the range where the greatest agreement is found between both scales.

The comparison reported in our study between the SCORE and D'Agostino scales at the cut-off point of high CVR of 20% in hypertensive patients aged 40–65 years with no cardiovascular history has not previously been performed. This is the first analysis to compare two mortality and morbidity and mortality scales measuring CVR. This is probably the reason why agreement between both scales shows a kappa index of 0.52, markedly better than reported by other authors comparing the Framingham-REGICOR and SCORE [19,21,27]or the Framingham and SCORE scales[12,13,27,28]. The reasons for the poorer agreement observed in previous studies may probably be that while the Framingham function estimates coronary morbidity and mortality, the SCORE equation estimates cardiovascular mortality.

Among patients classified as high CVR, approximately 9 out of every 10 subjects in whom discrepancies were found had high CVR according to the D'Agostino equation and non-high CVR according to SCORE, and only 1 had non-high CVR and high CVR using the D'Agostino and SCORE equations respectively. These figures, while substantial, are lower than the discrepancies reported in other studies[12,20].

The profiles of discordant patients classified as having a high CVR were different. Patients with high CVR in SCORE and non-high CVR according to D'Agostino, the least common profile, were older and had diabetes and a lower atherogenic index. By contrast, patients with high CVR according to D'Agostino and a non-high CVR using SCORE were males with a higher atherogenic index. Different profiles have been reported by other authors[12,13,20,21], all of whom agreed that the Framingham equation assigns a high CVR to a greater number of males.

From the data obtained, it can be concluded that males with an unfavourable lipid profile and non-high CVR in the SCORE scale probably show a CVR higher than estimated with this scale, which should be considered by clinicians when making therapeutic decisions.

Finally, use of the D'Agostino equation would imply treating more patients (particularly males) with lipid lowering drugs. This agrees with the conclusion drawn by Maiques et al [13] that SCORE promotes treatment in hypertensive females and Framingham in dyslipidaemic males.

Study limitations include the fact that the patient sample studied was not randomly taken from the general population, but consisted of consecutive patients with clinical hypertension referred to the research unit for ABPM for different reasons, mainly to confirm diagnosis, but part of the patients were already on antihypertensive and lipid lowering treatment, which may alter estimation of their cardiovascular risk. However, such limitations do not invalidate the comparison of both equations, because since all patients were assessed by both methods at the same time, the design used was adequate for assessing scale agreement without interfering with the comparison. The primary objective was to estimate and compare the CVRs inferred from both scales, as well as the prognostic and therapeutic implications depending on the result obtained. The scales are only an approach to the actual risk for the subject and this paper intends to contrast both risk estimation tools, though we are aware of the limitations of the instruments, as evidenced in the paper. We have been used as reference SCORE, recommend by Spanish and European regulatory authorities, because there aren't gold standard scale, to compare the new D'Agostino scale.

We have excluded from the analysis a very important population group where the prevalence of hypertension is very high. The reasons for selecting this population group is that the SCORE scale has been developed for a population aged 40 to 65 years, and though the D'Agostino scale allows for using it in a larger population group, the results of the SCORE in the elderly could limit the validity of the analyses performed.

## Conclusion

Discrepancies exist between the SCORE scale for low CVR countries and the D'Agostino equation with regard to both CVR assessment and identification of high CVR. Agreement improves when a cut-off point for high CVR of 29% instead of 20% is used in D'Agostino scale. The finding of discordant patient profiles may allow for a better approach to the clinical assessment of CVR and for identification of patients who, while having a non-high CVR according to SCORE, may have an actual CVR higher than estimated. Patients in this group would be candidates to lipid lowering treatment.

## Abbreviations

CVR: Cardiovascular risk; AH: Arterial hypertension, SCORE: Systematic Coronary Risk Evaluation; ABPM: Ambulatory blood pressure monitoring; SBP: Systolic blood pressure; DBP: Diastolic blood pressure; BMI: Body mass index; HDL-C: High-density lipoprotein cholesterol; LDL-C: Low-density lipoprotein cholesterol (LDL-C).

## Competing interests

The authors declare that they have no competing interests.

## Authors' contributions

MAGM devised the study, designed the protocol, participated in fund raising, and prepared the draft manuscript. CMS and ERS collaborated in protocol design, data interpretation, and review of the manuscript. JCMC and MGC participated in study design, interpretation of results, and manuscript review. JIRR and YCS participated in study design, data collection, and manuscript review. LGO participated in protocol design, fund raising, analysis of results, and final review of manuscript. Finally, all authors reviewed and approved the final version of the manuscript

## Pre-publication history

The pre-publication history for this paper can be accessed here:


